# Comparative transcriptome analysis of two selenium-accumulating genotypes of *Aegilops tauschii* Coss. in response to selenium

**DOI:** 10.1186/s12863-018-0700-1

**Published:** 2019-01-14

**Authors:** Lijun Wu, Tao Liu, Yongsheng Xu, Wenjie Chen, Baolong Liu, Lianquan Zhang, Dengcai Liu, Huaigang Zhang, Bo Zhang

**Affiliations:** 1 Key Laboratory of Adaptation and Evolution of Plateau Biota (AEPB), Northwest Institute of Plateau Biology, Chinese Academy of Sciences, 23# Xinning Lu, Xining, 810008 Qinghai China; 2 Qinghai Provincial Key Laboratory of Crop Molecular Breeding, Xining, 810008 China; 3 University of Chinese Academy of Sciences, Beijing, 100049 China; 4 Triticeae Research Institute, Sichuan Agricultural University, Chengdu, 611130 China; 5 Xining Administration Center of Parks, Xining, 810001 China

**Keywords:** Differentially expressed genes, Selenium, *Aegilops tauschii*

## Abstract

**Background:**

Selenium (Se), an essential micronutrient in both animals and humans, has various biological functions, and its deficiency can lead to various diseases. The most common method for increasing Se uptake is the consumption of Se-rich plants, which transform inorganic Se into organic forms. Wheat is eaten daily by many people. The Se content of *Aegilops tauschii (Ae. tauschii)*, one of the ancestors of hexaploid common wheat, is generally higher than that of wheat. In this study, two genotypes of *Ae. tauschii* with contrasting Se-accumulating abilities were subjected to different Se treatments followed by high-throughput transcriptome sequencing.

**Results:**

Sequencing of 12 transcriptome libraries of *Ae. tauschii* grown under different Se treatments produced about a total of 47.72 GB of clean reads. After filtering out rRNA sequences, approximately 19.3 million high-quality clean reads were mapped to the reference genome (ta IWGSC_MIPSv2.1 genome DA). The total number of reference genome gene is 32,920 and about 26,407 known genes were detected in four groups. Functional annotation of these mapped genes revealed a large number of genes and some pivotal pathways that may participate in Se metabolism. The expressions of several genes potentially involved in Se metabolism were confirmed by quantitative real-time PCR.

**Conclusions:**

Our study, the first to examine Se metabolism in *Ae. tauschii*, has provided a theoretical foundation for future elucidation of the mechanism of Se metabolism in this species.

**Electronic supplementary material:**

The online version of this article (10.1186/s12863-018-0700-1) contains supplementary material, which is available to authorized users.

## Background

Selenium (Se) is an essential trace element with a variety of biological functions [1, 2], and its deficiency can cause a series of health problems [3]. Se in wheat grains has high bioavailability [4]. Bread wheat (*Triticum aestivum* L., genome AABBDD), a major staple crop worldwide, is humanity’s main food source. Bread wheat is an allohexaploid that originated from hybridization between cultivated tetraploid wheat (*T. turgidum* L., BBAA) and the diploid wheat relative *Aegilops tauschii* Coss. (DD). A previous study has indicated that *Aegilops tauschii* (*Ae. tauschii*) possesses a higher ability to accumulate Se than does bread wheat [5].

The final content of Se in plants is controlled by three processes: uptake of Se from soil, assimilation of Se, and translocation into different organs. An understanding of the molecular mechanisms of Se uptake, assimilation, and translocation would thus facilitate the development of cereal crops with increased Se grain contents [6]. One important tool to aid elucidation of these mechanisms is RNA-seq technology*.*

In the present study, the transcriptome sequences of two *Ae. tauschii* accessions with different Se-accumulating abilities grown under various Se treatments were generated by RNA-seq, de novo assembled, and annotated. Comparative transcriptome analysis of leaves of the two genotypes was performed to reveal the differences in their transcriptional responses to Se treatment and to determine the genetic basis of their different Se accumulation capabilities. To improve understanding of Se metabolism in *Ae. tauschii* [7], bioinformatic tools were applied to investigate the pathways and genes expressed under Se treatment. The results of this study provide new insights into the molecular mechanisms underlying Se accumulation in *Ae. tauschii*, which should aid the development of new, more efficient methods to improve the Se contents of *Ae. tauschii*, or even wheat via molecular breeding.

## Results

For use in this study, we selected *Ae. tauschii* ssp*. strangulata* AS2407 and *Ae. tauschii* ssp*. tauschii* AS65, which have the highest and lowest Se levels, respectively, of 10 different frequently used accessions [8]. To test the effect of Se on *Ae. tauschii*, pre-treated plants were cultivated hydroponically in plastic containers containing quarter-strength modified Hoagland’s nutrient solution under a continuous oxygen supply.

### RNA sequencing of leaf transcriptomes of the two genotypes

RNA-seq followed by strict quality control and processing generated a total of 47.72 GB of clean data from 12 transcriptome libraries. The 12 transcriptome libraries represented four treatment groups with three repetitions: CK1 (AS65 treated with water), CK2 (AS2407 treated with water), T1 (AS65 treated with 10 μM Na_2_SeO_3_), and T2 (AS2407 treated with 10 μM Na_2_SeO_3_). After filtering out duplicate sequences and ambiguous and low-quality reads, an average of 31.42 × 10^6^ high-quality (HQ) clean reads remained per library: 32.14 million for CK1, 29.68 million for CK2, 31.98 million for T1, and 33.46 million for T2. Excluding rRNA sequences, this corresponded to 27.54 to 30.95 million High-quality clean reads (HQ clean reads) per group (Table 1; see Additional file 1: Data S2 for details). The average GC percentage was 57.83%, with a QC30 base percentage above 95.45%. Details on data and data quality before and after filtering are shown in Additional file 2: Figure S1 and Additional file 3: Data S1. All transcriptome data are shown in Table 1.

**Table 1:** Sequencing and statistics for the four transcriptome data after Se treatment

Group name	CK1	CK2	T1	T2
No. of Clean Reads (×10^6^)	32.14 ± 6.72	29.68 ± 4.38	31.98 ± 0.90	33.46 ± 2.94
No. of HQ Clean Reads (×10^6^)	31.75 ± 6.67	29.31 ± 4.31	31.58 ± 0.91	33.03 ± 2.89
rRNA Mapped Reads	1.06 ± 0.57	1.76 ± 0.56	0.63 ± 0.17	2.12 ± 1.06
No. of HQ clean reads except the rRNA (×10^6^)	30.69 ± 6.27	27.54 ± 4.43	30.95 ± 0.99	30.90 ± 3.13

No. is short for number

### Mapping of RNA sequences to the reference genome

HQ clean reads, except for rRNA, were mapped to the *Ae. tauschii* reference genome (ta IWGSC_MIPSv2.1 genome DA). Approximately 19.3 million clean reads (64.34% of the total) were mapped; 0.33 million were multiply mapped, and 18.97 million were unique. An overview and detailed data are given in Table 2 and Additional file 4: Data S3.

**Table 2:** Sequencing and statistics for the four group’s transcriptome data with the reference genome (ta IWGSC_MIPSv2.1 genome DA)

Group name	CK1	CK2	T1	T2
No. of total reads (×10^6^)	30.69 ± 6.27	27.54 ± 4.43	30.95 ± 0.99	30.90 ± 3.13
No. of mapped reads (×10^6^)	19.16 ± 4.02	18.24 ± 3.00	19.38 ± 0.59	20.42 ± 1.90
Mapped percentage	62.48% ± 1.23%	66.21% ± 0.46%	62.63% ± 0.50%	66.10% ± 1.02%
Unique Mapped Reads (×10^6^)	18.84 ± 3.95	17.94 ± 2.95	19.04 ± 0.57	20.06 ± 1.86
Multiple Mapped reads (×10^6^)	0.32 ± 0.08	0.30 ± 0.05	0.34 ± 0.02	0.36 ± 0.05

### Gene expression statistics

The total number of reference genome gene is 32,920 and about 26,407 known genes (about 80.22%) were detected in four groups. New genes accounted for 19.66% of the total. The number of genes in each group and sample is summarized in Table 3 and Additional file 5: Data S4, respectively.

**Table 3:** Detection gene number statistics for each group

Group name	Known gene num	New gene num	All gene num
CK1	23,803 (72.31%)	5611	29,414
CK2	24,271 (73.73%)	5719	29,990
T1	23,515 (71.43%)	5589	29,104
T2	24,311 (73.85%)	5745	30,056

To check the reliability of the expression data, we compared the results among replicates. Pairwise Pearson correlation coefficients were calculated between replicates and represented as a heat map, with the intensity of the correlation indicated with a color scale. As can be seen on the correlation heat map of Additional file 6: Figure S2, the expression data of CK1–3 was not only very different from the other two replicates, but also the other groups. Because this aberrant expression indicates that the CK1–3 sample was contaminated or damaged, the CK1–3 data were not used in subsequent analyses.

A comparison of changes in gene expression between different groups revealed considerable differences. As a result of deep sequencing of the four groups, more than 27,000 genes (82.02%) in the *Ae. tauschii* reference genome were detected in each group. The samples exhibited transcriptional complexity in terms of the different genotypes and Se treatments.

### Distribution of expressed genes

**Fig. 1:**
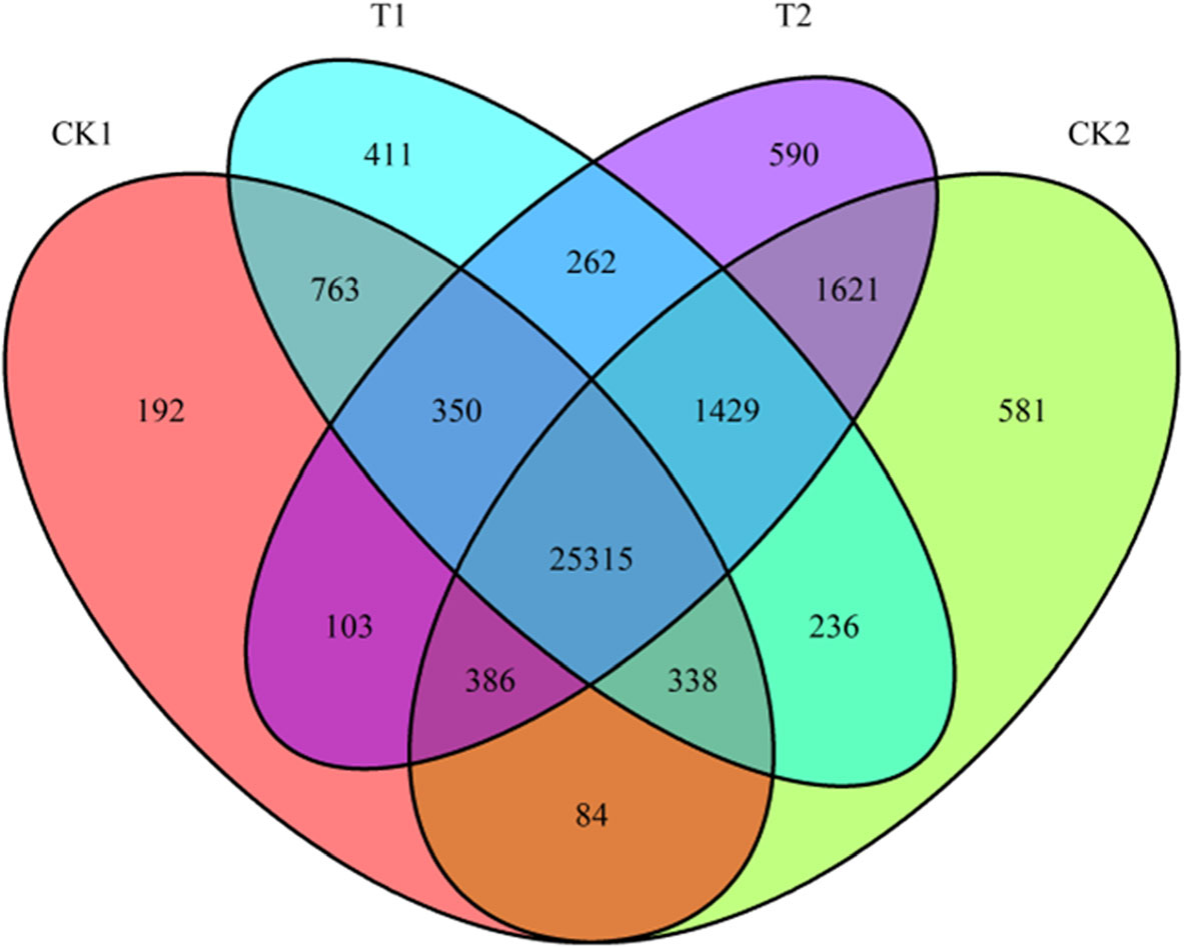
The Venn diagram is exhibiting the overlaps in four groups

Venn diagram analysis of *Ae. tauschii* genes coexpressed in the four groups revealed 26,123 coexpressed in CK1 and CK2, 26,776 in both CK1 and T1, 28,751 in both CK2 and T2, 27,356 in both T1 and T2. The number of specifically expressed genes in each group was as follows: 192 (CK1), 411 (CK2), 590 (T1), and 581 (CK2) (Fig. 1; see Additional file 7: Data S5 for a list of genes by category). In addition, 25,315 genes were coexpressed in all four groups. Some genes, probably related to housekeeping functions, exhibited little variation across different groups, while others were quantitatively regulated. The dynamic nature of gene expression in the four samples suggests that the different genotypes and Se treatments affected the absorption of Se in *Ae. tauschii*.

To obtain statistical confirmation of differences in gene expression among the four groups, we normalized gene expression levels using the Fragments Per Kilobase of exon model per Million mapped reads (FPKM) method. The expressions of genes were classified according to their calculated FPKMs as low (0–10), medium (10–50), or high (> 1000). The greatest proportion of genes in each group was those lowly expressed, with the smallest number expressed at a high level (Table 4). With or without Se treatment, more genes were expressed in AS2407 than AS65. Although the relationship trend between the number and percentage of expressed genes was consistent between genotypes, little difference was observed between Se and control treatments within a given genotype. In the present study, genotype had a more obvious effect on gene expression than exposure to 10 μM Na_2_SeO_3_.

**Table 4:** Statistics of genes in different expression-level interval

FPKM interval	CK1 (%)	CK2 (%)	T1 (%)	T2 (%)
0–10	16,836 (61.15)	18,187 (60.64)	18,326 (62.97)	18,486 (61.51)
10–50	7600 (19.88)	8414 (20.13)	7791 (19.53)	8184 (19.66)
50–100	1635 (5.34)	1770 (5.30)	1565 (4.88)	1735 (5.19)
100–500	1220 (4.21)	1381 (4.37)	1194 (3.91)	1407 (4.44)
500–1000	125 (0.45)	139 (0.46)	117 (0.4)	139 (0.46)
>1000	115 (0.42)	99 (0.33)	112 (0.38)	105 (0.35)

### Analysis of differentially expressed genes (DEGs)

**Fig. 2:**
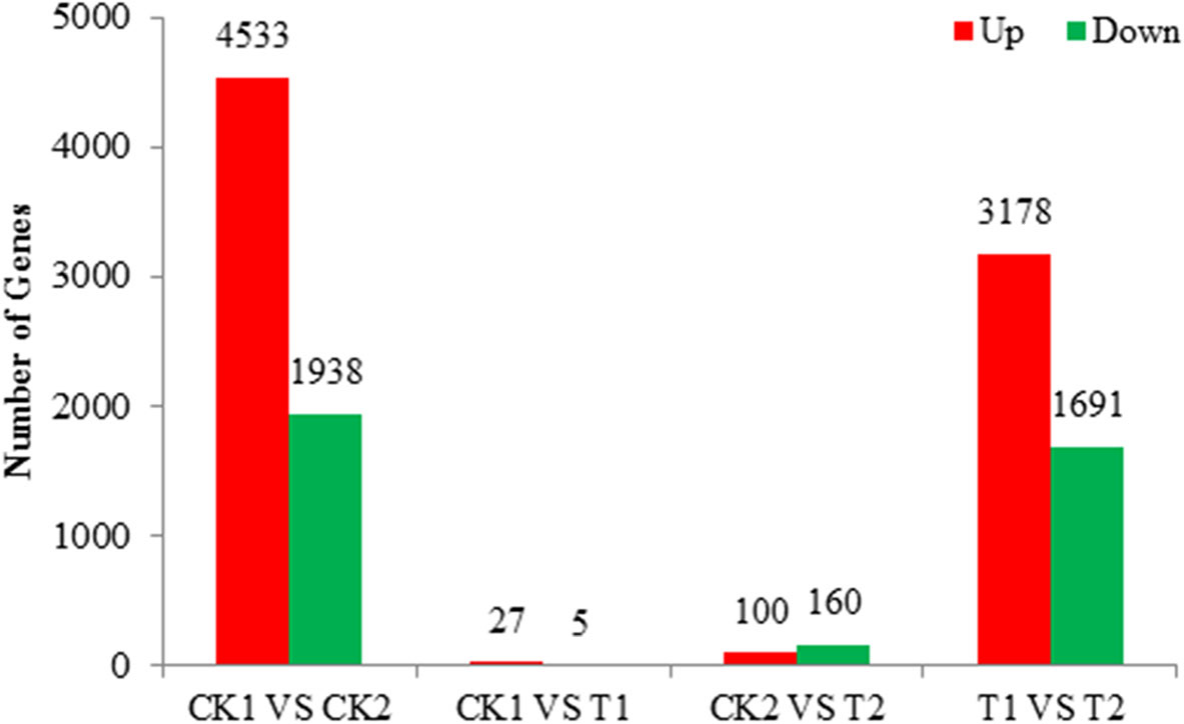
Changes in gene expression profile between different groups

To uncover significantly expressed genes between groups, genes differentially expressed between the two genotypes after 48 h under different Se conditions were identified using edgeR software with a false discovery rate (FDR)-adjusted *p*-value threshold [9, 10]. Transcripts with at least a two-fold difference in expression (|log_2_ fold change| > 1) and a FDR < 0.05 were considered to be differentially expressed between groups [11]. In the absence of Se, 5052 genes were differentially expressed between CK1 and CK2, among which 4533 were up-regulated and 1938 were down-regulated in CK2 compared with CK1 (CK1 vs. CK2). Under 10 μM Se, we detected 4315 DEGs in T1 vs. T2, 2856 up-regulated and 1459 down-regulated. In AS65, 32 DEGs (27 up-regulated and 5 down-regulated) were expressed in CK1 vs. T1, while 322 DEGs (132 up-regulated and 140 down-regulated) were detected between CK2 and T2 in AS2407 (Fig. 2). These results suggest that the number of DEGs between genotypes were more than those between Se treatments. At 10 μM Na_2_SeO_3_, the maximum concentration that can safely be applied to *Ae. tauschii*, the influence of added Se was much smaller than that of genotype.

**Fig. 3:**
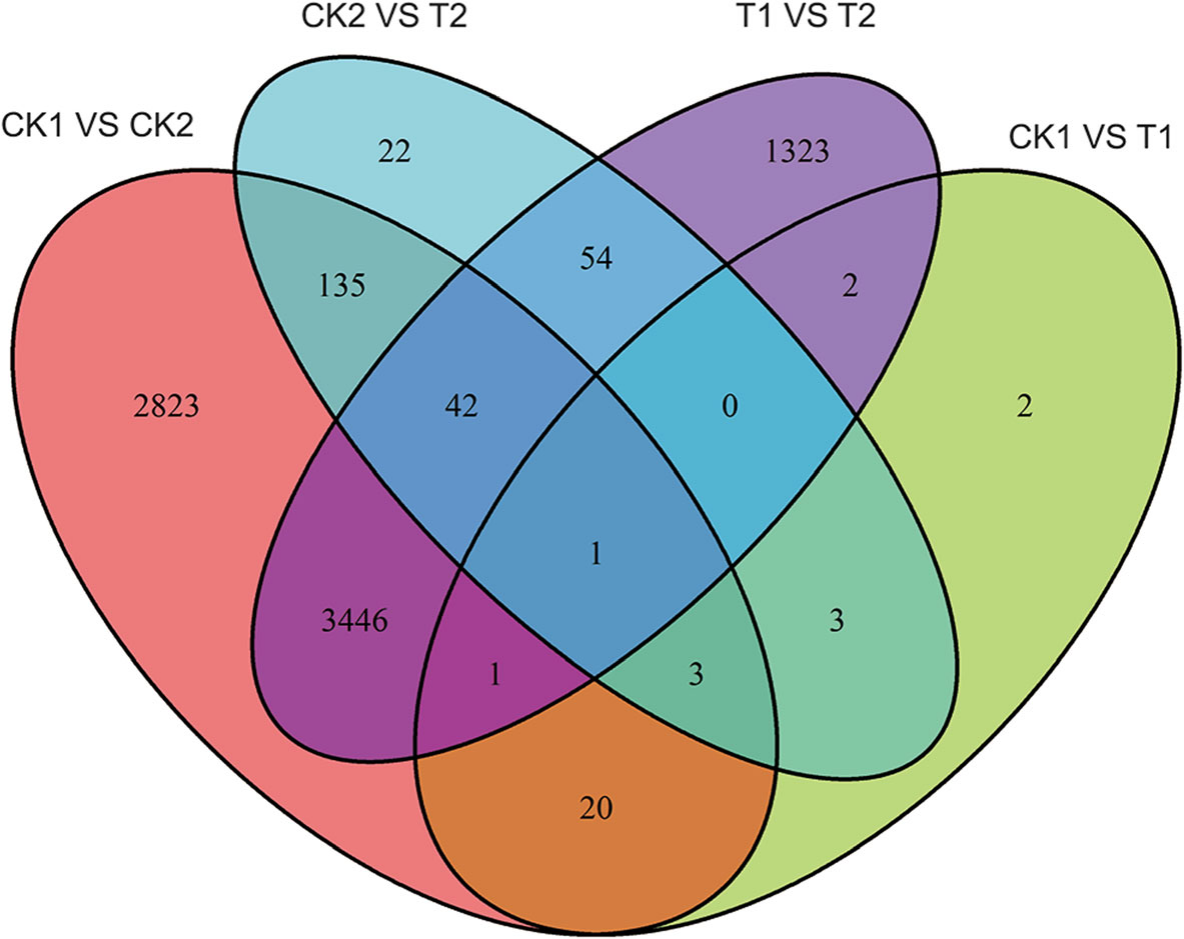
The Venn diagram shows the differentially expressed genes between each two groups and the number of overlapping expression of genes

An RNA-seq approach was used to preliminarily identify and analyze the differential expression of *Ae. tauschii* genes associated with Se. Because the two different genotypes pretreated with Se exhibited obvious changes in gene expression, DEGs between the four treatment groups were examined more extensively (Fig. 1). Among these DEGs, seven genes encoding the following proteins were regulated in an Se-related manner (Fig. 3): disease resistance protein RGA2-like (predicted, gene ID: XLOC_024936), Auxilin-related protein 1 (gene ID: XLOC_034135), protein transport protein SEC23-like (gene ID: XLOC_038938), Hessian fly response gene 1 protein (gene ID: XLOC_046021), Chalcone synthase 1 (gene ID: Traes_2DL_BC7F606B9), Fatty acyl-CoA reductase 1 (gene ID: Traes_5DL_5597A11EC) and beta-amylase (gene ID: Traes_2DS_D14ABD1DB). The seven proteins known to interact with Se were verified by real-time fluorescence quantitative PCR (RT-qPCR) (Additional file 8: Figure S3). There is a certain error that the fold changes were very high because the FPKM of one simple was almost zero and another simple was less than 10 in the RNA-seq data sequencing. This happened in gene XLOC_024936 and XLOC_034135. As indicated by the general correlation coefficient (*R*^*2*^ = 0.9275) (exclusive of gene XLOC_024936 and XLOC_034135), a good concordance was observed between the expression patterns of DEGs obtained by RNA-seq and RT-qPCR data. Chalcone synthase 1 is involved in flavonoid and secondary metabolite biosynthetic pathways. Genes regulating secondary metabolite biosynthesis are interesting candidates for further physiological and molecular investigations of Se stress tolerance in *Ae. tauschii*.

### Functional analysis of Se-influenced genes

**Fig. 4:**
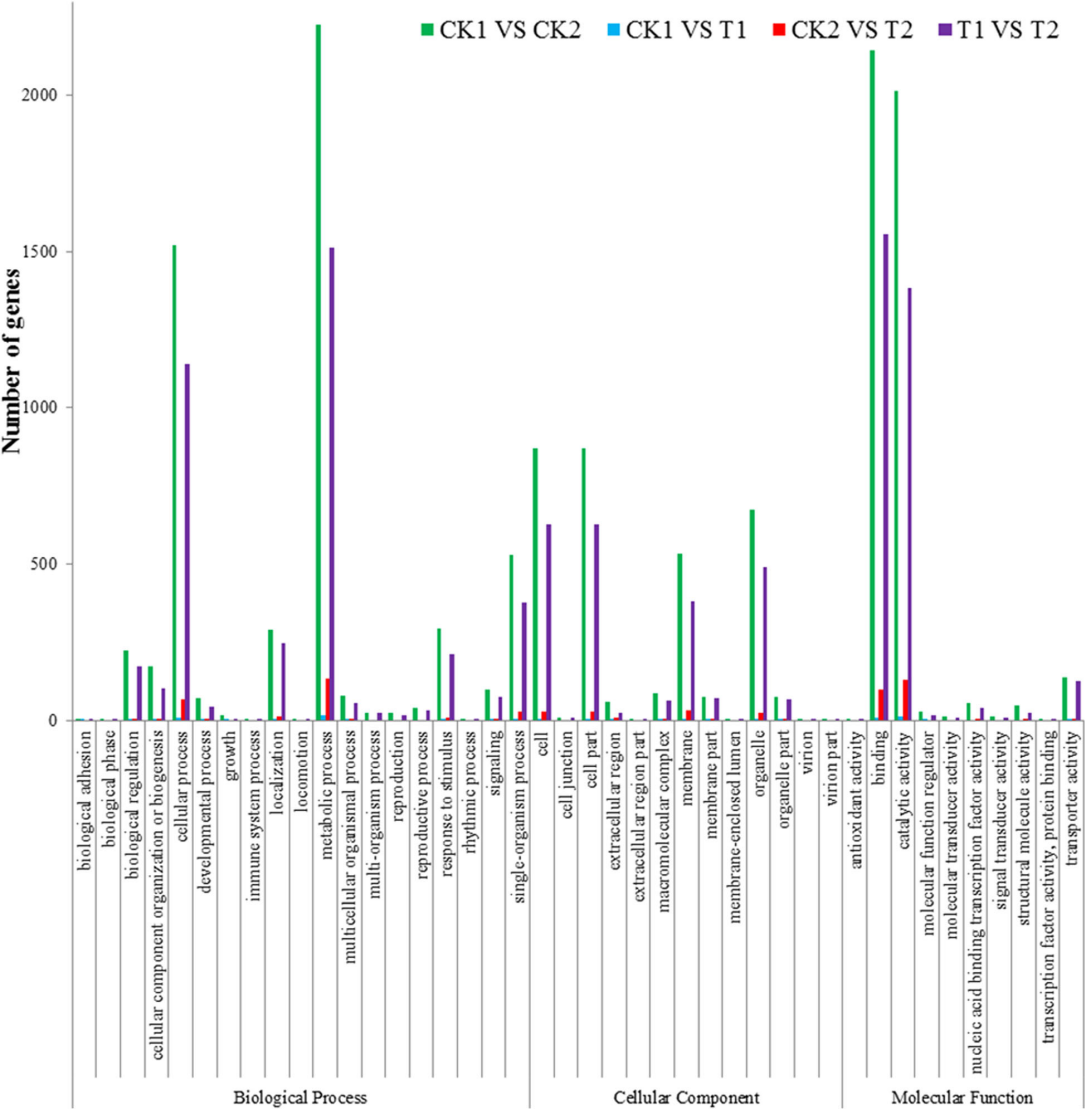
Histogram of GO enrichment terms as biological process, cellular component and molecular function of four groups

To facilitate global analysis of gene expression, we investigated the possible mechanisms of significant DEGs by Gene Ontology (GO) analysis (Fig. 4). The 13,354, 99, 635, and 9775 significant (q-value < 0.05) DEGs in CK1 vs. CK2, CK1 vs. T1, CK2 vs. T2, and T1 vs. T2, respectively, were categorized into 42, 24, 23, and 42 GO terms in the three main GO categories of biological process, cellular component, and molecular function (Fig. 4). DEG- enriched GO terms in CK1 vs. CK2 and T1 vs. T2 were identical. The mostly highly enriched GO terms in CK1 vs. CK2, CK1 vs. T1, CK2 vs. T2, and T1 vs. T2 were catalytic activity (GO: 0003824; 2014, 14, 129, and 1382 genes, respectively, binding (GO: 0005488; 2144, 10, 97, and 1556 genes), cellular process (GO: 0009987; 1520, 8, 67, and 1141 genes), and metabolic process (GO: 0008152; 2227, 16, 134, and 1512 genes). The genes in these different expression clusters associated with different GO terms clearly indicate biological processes and molecular functions that are Se-related.

Kyoto Encyclopedia of Genes and Genomes (KEGG) pathway analysis was used to assign 1136 DEGs in CK1 vs. CK2 to 125 pathways, 14 in CK1 vs. T1 to 22 pathways, 60 in CK2 vs. T2 to 64 pathways, and 740 in T1 vs. T2 to 121 pathways. The most obviously enriched metabolic pathways (*q*-value ≤0.05) in CK1 vs. CK2, CK2 vs. T2, and T1 vs. T2 respectively totaled 16, 8, and 6. Cutin, suberin and wax biosynthesis (ko00073) and flavonoid biosynthesis (ko00941) were the most common obviously enriched metabolic pathways in CK1 vs. CK2 and CK2 vs. T2, while the following pathways were especially enriched in CK2 vs. T2: anthocyanin biosynthesis (ko00942), beta-alanine metabolism (ko00410), pyruvate metabolism (ko00620), tyrosine metabolism (ko00350), isoquinoline alkaloid biosynthesis (ko00950), and ascorbate and aldarate metabolism (ko00053). In CK1 vs. CK2 and T1 vs. T2, the most common highly enriched metabolic pathways were plant–pathogen interaction (ko04626), glutathione metabolism (ko00480), fructose and mannose metabolism (ko00051), phenylpropanoid biosynthesis (ko00940), and aminoacyl-tRNA biosynthesis (ko00970). Phenylalanine metabolism (ko00360) was the only obviously enriched metabolic pathway in T1 vs. T2. Although flavonoid biosynthesis was an enriched metabolic pathway in each comparison group, its *q*-value was greater than 0.05 in CK1 vs. T1 and T1 vs. T2.

For elucidating the useful information related to selenium accumulation, expressed genes in each group of CK1 vs. T1 and CK2 vs. T2 were compared. The 83 and 619 unique DEGs in CK1 vs. T1 and CK2 vs. T2 were respectively categorized into 24 and 24 GO terms in the three main GO categories of biological process, cellular component, and molecular function (Additional file 9: Data S6). Interestingly, the unique DEGs in the two groups were categorized into the same 21 GO terms related to cellular process, metabolic process, membrane, catalytic activity, transporter activity, and so on. The 10 and 65 unique DEGs in each CK1 vs. T1 and CK2 vs. T2 were respectively categorized to 17 and 63 pathways, among them 10 pathways were common (Additional file 10: Data S7). ABC transporters, glutathione metabolism, Phenylalanine metabolism, Circadian rhythm – plant, Flavonoid biosynthesis, and Glycolysis / Gluconeogenesis were found in unique CK2 vs. T2.

### Experimental validation of DEGs by RT-qPCR

**Fig. 5:**
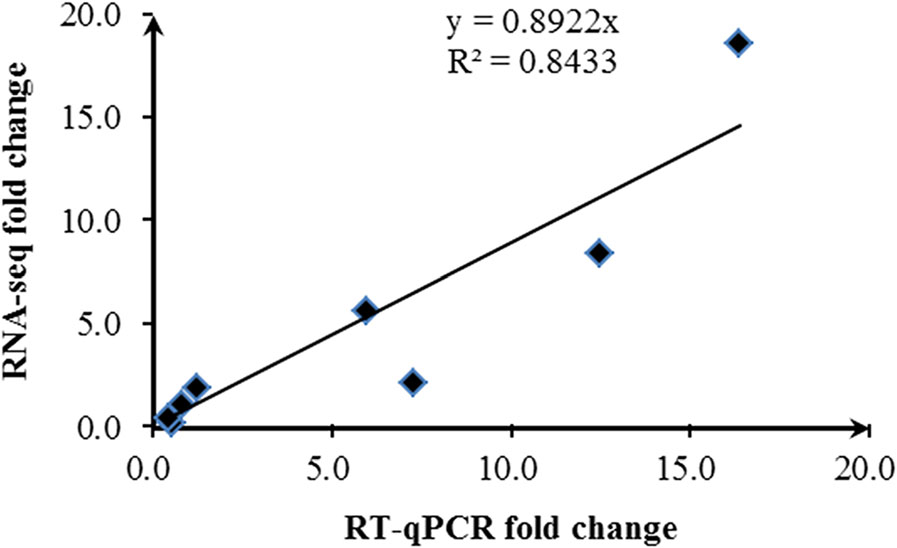
Correlation of fold change values between RNA-seq and RT-qPCR. The *R*^*2*^ value is 0.8433

To evaluate the validity of the RNA-seq data, we carried out a RT-qPCR analysis of eight DEGs involved in flavone and flavonol biosynthesis [7], flavonoid biosynthesis [7], and selenocompound metabolism which were believed to be related to selenium metabolism. As shown in Fig. 5, the expression patterns determined by RT-qPCR were in agreement with those based on RNA-seq data. As indicated by the general correlation coefficient (*R*^*2*^ = 0.8433), a good concordance was observed overall between the expression patterns of DEGs obtained by RNA-seq and RT-qPCR data, thus supporting the reliability of the data.

### Transcription factors (TFs) related to Se

Many putative TF genes, including bHLH, WRKY, MYB, and AP2 (Table 5 and Additional file 11: Data S8), were identified by querying the Plant Transcription Factor Database (http://planttfdb.cbi.pku.edu.cn/). The expression trends of differentially expressed TFs in CK1 vs. CK2 were consistent with those common to T1 vs. T2; the same was true between CK1 vs. T1 and CK2 vs. T2. Most of the common TFs were down-regulated. M-type MADS genes were all down-regulated in CK1 vs. T1 and CK2 vs. T2—in other words, after Se treatment. Genes encoding AP2 proteins were down-regulated in CK1 vs. CK2 and T1 vs. T2, while a gene (Traes_5DL_32D78D06A) encoding the WRKY TF was up-regulated in all groups.

**Table 5:** Common difference expression transcription factors in four groups

Group	Gene ID	Description	CK1 FPKM	CK2 FPKM	T1 FPKM	T2 FPKM	Fold change			
							CK1 VS CK2	CK1 VS T1	CK2 VS T2	T1 VS T2
bHLH	XLOC_013854	Transcription factor PIF3	12.33	42.66	15.73	73.21	3.46	1.28	1.72	4.65
	Traes_2DL_865346DA2	Transcription factor ORG2	73.52	19.92	67.07	17.30	0.27	0.91	0.87	0.26
	XLOC_014379	Transcription factor bHLH93	14.73	3.90	15.19	3.31	0.26	1.03	0.85	0.22
WRKY	Traes_5DL_32D78D06A	WRKY transcription factor	2.70	77.10	5.04	112.02	28.56	1.87	1.45	22.23
MYB	Traes_7DL_090D0E08E	Myb family transcription factor APL	15.45	33.52	15.41	36.98	2.17	1.00	1.10	2.40
	XLOC_006616	Myb family transcription factor APL	44.25	5.61	30.46	4.96	0.13	0.69	0.88	0.16
M-type MADS	Traes_3DL_915546ED4	MADS-box transcription factor TaAGL41	25.23	59.11	23.01	48.34	2.34	0.91	0.82	2.10
	Traes_4DL_C4CB3D5AF	MADS-box transcription factor TaAGL33	27.75	62.73	26.11	54.00	2.26	0.94	0.86	2.07
	Traes_3DL_FBB80151F	MADS-box transcription factor TaAGL42	26.65	1.51	26.76	1.42	0.06	1.00	0.94	0.05
AP2	XLOC_004436	AP2-like ethylene-responsive transcription factor ANT	15.32	0.42	19.20	0.85	0.03	1.25	2.02	0.04
	XLOC_028083	AP2-like ethylene-responsive transcription factor ANT	41.35	2.82	50.86	3.36	0.07	1.23	1.19	0.07
	XLOC_040922	AP2/EREBP type transcription factor	43.00	3.70	28.89	5.81	0.09	0.67	1.57	0.20

Except for common TFs, TFs in each comparison group (Additional file 11: Data S8), including NAC, WRKY. and bHLH, were mostly up-regulated. MYB TFs in CK1 vs. T1 and in CK2 vs. T2 were down- regulated, whereas bHLH TFs in CK1 vs. CK2 and in T1 vs. T2 were all up-regulated.

## Discussion

Selenium is an essential trace mineral of fundamental importance to human health [12]. Humans rely on plants, including cereal crops, as a medium to supplement nutritional Se. The Se hyperaccumulator *Astragalus bisulcatus* and the secondary Se accumulator *Brassica oleracea var. italica* (broccoli) are well-known models for investigating the molecular and biochemical basis of Se in plants [13]. In contrast, studies related to Se metabolism in Triticeae crops have not been reported.

The advent of RNA-seq has simplified the acquisition of transcriptome data from plant tissues or cells under specific conditions [7]. In the present study, we thus aimed to elucidate the molecular basis of Se metabolism in *Ae. tauschii* by RNA-seq. A total of 47.72 GB of clean data were generated, with 32.14, 29.68, 31.98, and 33.46 million clean reads respectively obtained from CK1, CK2, T1, and T2 samples after filtering out low-quality reads. Approximately 19.3 million clean reads (64.34% of the total) were mapped to the *Ae. tauschii* reference genome (ta IWGSC_MIPSv2.1 genome DA). A total of 26,407 known genes were detected in the four treatment groups, with unidentified genes accounting for 19.66% of genes inferred from the reference genome. We uncovered 25,315 genes coexpressed in all four groups, while some genes were coexpressed in two or three groups (Fig. 1). Statistical confirmation of gene expression differences among the four groups demonstrated that genotype was a more obvious influence than treatment with 10 μM Na_2_SeO_3_. Analysis of DEGs in the four groups further strengthened the observation that the effect of Se concentration was much smaller than that of genotype. The relatively inconsequential effect of added Na_2_SeO_3_ may be mainly because a concentration of 10 μM was insufficient to counterbalance the differences between genotypes. Co-DEGs in CK1 vs. T1 and CK2 vs. T2 were verified and a good concordance (*R*^*2*^ = 0.9275) was observed between the expression patterns of DEGs obtained by RNA-seq and RT-qPCR data, thus supporting the reliability of the data. Genes regulating secondary metabolite biosynthesis, such as chalcone synthase 1 preliminarily identified by analysis of Se-associated DEGs, are interesting candidates for further physiological and molecular investigations of Se stress tolerance in *Ae. tauschii*. The five co-DEGs (protein transport protein SEC23-like, Hessian fly response gene 1 protein, Chalcone synthase 1, Fatty acyl-CoA reductase 1 and beta-amylase) will be the potential candidates for Se related function analysis in our future study. GO enrichment analysis of significant DEGs revealed the biological processes and molecular functions involved in Se absorption. Flavonoid biosynthesis was the most common obviously enriched metabolic pathway in AS2407 vs. AS65 (both treated with water) and Se-treated vs. water-treated AS2407. Metabolites possibly useful for plant adaptation to the environment are thought to be produced via flavone and flavonol biosynthesis, flavonoid biosynthesis pathways [7]. In addition, phenylalanine metabolism (ko00360) was an especially highly enriched metabolic pathway in Se-treated AS2407 compared with Se-treated AS65. DEGs associated with flavonoid biosynthesis and phenylalanine metabolism should be further explored in future research related to Se. ABC transporters, Phenylalanine metabolism, Circadian rhythm – plant, Flavonoid biosynthesis, and Glycolysis / Gluconeogenesis enriched in unique CK2 vs. T2 compared with CK1 vs. T1 which were consistent with Özgür Çakır [7] were be regarded as related to the metabolism of selenium. Selenium is an important component of glutathione peroxidase in glutathione metabolism which was also found in unique CK2 vs. T2 compared with CK1 vs. T1. Se treatment made the above pathways responded in AS2407 (T2). The unique DEGs in CK2 vs. T2 may be the reason why the content of Se in AS2407 was higher than in AS65.

Eight DEGs involved in flavone and flavonol biosynthesis, flavonoid biosynthesis, and selenocompound metabolism was performed by RT-qPCR to evaluate the validity of the RNA-seq data, the result was in agreement. But a small amount of difference was uncovered, and it may be caused by differences in the algorithms used to determine expression levels [14].

All major life processes depend on differential gene expression, a phenomenon largely controlled by TFs [15]. Many TF families may play important roles in developmental processes [16] in which TFs can act as activators, repressors, or both [15]. Se treatment may cause aberrant expression of many protein-coding genes [7]. M-type MADS proteins are involved the control of flower development in plants [17], while AP2 encoding proteins participate in the regulation of processes such as flower and embryo development [18]. WRKY proteins are a superfamily of TFs [19] that play important roles in responses to abiotic stress [20–22]. These TFs are related to plant development and respond to a variety of stresses [23]. In our study, WRKY genes were mostly up-regulated under Se treatment, a result in agreement with the finding that selenite treatment up-regulates genes encoding the WRKY TF family in *Astragalus chrysochlorus* [24]. MYB genes in CK1 vs. T1 and CK2 vs. T2 were down-regulated in our study; this is contrary to the results of Agarwal et al. [25], but different concentrations and chemical forms of Se were used in their study. The bHLH TFs can regulate flavonoid, alkaloid, and terpene biosynthesis [26, 27], which is consistent with our results. Flavonoid biosynthesis, which is somewhat related to environmental adaptation and stress response, is affected by Se treatment [7]. bHLH TF genes differentially expressed between the two genotypes in our study are likely associated with Se treatment.

## Conclusions

In this study, we used gene expression data to analyze differences in transcription-level responses of two genotypes of *Ae. tauschii* to Se. The number of DEGs in Se- vs. water-treated AS2407 was larger than in similarly treated AS65 plants, a finding consistent with the greater Se-absorbing ability of AS2407 vs. AS65 [8]. The five co-expression genes (protein transport protein SEC23-like, Hessian fly response gene 1 protein, Chalcone synthase 1, Fatty acyl-CoA reductase 1 and beta-amylase) in CK1 vs. T1 and CK2 vs. T2 and eight DEGs involved in flavone and flavonol biosynthesis, flavonoid biosynthesis, and selenocompound metabolism were believed to be potentially related to selenium metabolism. The pathways (ABC transporters, Phenylalanine metabolism, Circadian rhythm – plant, Flavonoid biosynthesis, and Glycolysis / Gluconeogenesis and glutathione metabolism) enriched by unique DEGs in CK2 vs. T2 compared with CK1 vs. T1 were regarded as related to the metabolism of selenium and it may be the reason why the content of Se in AS2407 was higher than in AS65. Finally, our results may help inform the interpretation of some TFs and metabolic modifications related to Se as all of these reactions may occur between the modifications in gene expression and the resulting physiological response.

## Methods

### Plant materials and Se treatments

*Ae. tauschii.* ssp. *strangulata* AS2407 and *Ae. tauschii* ssp. *tauschii* AS65 were selected for use in this study. Seeds of both accessions were obtained from Sichuan Agricultural University, Sichuan Province, China. The seeds were soaked in deionized water at room temperature for 24 h and then germinated on moist filter paper in Petri dishes at 24 ± 1 °C under a 16/8-h light/dark cycle. Two-week-old germinated seedlings were transplanted from the Petri dishes to plastic containers containing quarter-strength modified Hoagland’s nutrient solution in a greenhouse under the same temperature and photoperiod conditions [28]. The composition of the nutrient solution was as follows: 1.0 mM Ca(NO_3_)_2_·4H_2_O, 1.25 mM KNO_3_, 0.25 mM NH_4_NO_3_, 0.25 mM KH_2_PO_4_, 0.95 mM MgSO_4_, 1.25 μM KI, 36.75 μM MnSO_4_, 25 μMH_3_BO_3_, 13.35 μM ZnSO_4_, 0.25 μM Na_2_MoO_4_·2H_2_O, 0.0475 μM CuSO4, 0.05 μM CoCl_2_, and 12.5 μM Fe(III)-EDTA. After 40 days of growth, the plants were treated with fresh medium supplemented with Na_2_SeO_3_ to a final concentration of 10 μM, a level of Se that causes mild stress in *Ae. tauschii* but no observable toxic symptoms. After 72 h, young leaf samples from at least three similarly sized Se-treated or control plants of each genotype were harvested separately. Fresh leaf tissues were frozen in liquid nitrogen and stored at − 80 °C for RNA extraction.

### RNA extraction and RNA-Seq library construction and sequencing

Total RNA was separately extracted from frozen leaf tissues of three samples per treatment using a Trizol kit (Promega, USA) following the manufacturer’s protocol. The extracted RNA was treated with RNase-free DNase I (Takara Bio, Japan) for 30 min at 37 °C to remove residual DNA. The quality of the purified RNA was verified by agarose gel electrophoresis, and its concentration was measured on a 2100 Bioanalyzer (Agilent Technologies, Santa Clara, CA, USA) at 260 nm and 280 nm. High-quality RNA from the three sample replicates was combined for subsequent RNA sequencing.

The extracted RNA samples were used for cDNA synthesis. Poly (A) mRNA was isolated using oligo-dT beads (Qiagen) and broken into short fragments (200 nt) with fragmentation buffer. First-strand cDNA was generated by random hexamer-primed reverse transcription using the fragmented mRNA as a template, with second-strand cDNA then synthesized after addition of buffer, dNTPs, RNase H, and DNA polymerase I. The generated cDNA fragments were purified using a QIAquick PCR gel extraction kit (Qiagen, Germany), washed with EB, subjected to end reparation and poly (A) addition, and ligated to sequencing adapters. cDNA fragments of interest (200 ± 25 bp) were purified and enriched by PCR amplification to complete preparation of the entire cDNA library. The constructed cDNA library was sequenced in a single run on the IlluminaHiSeq 2500 platform using single-end and paired-end technology at Beijing Genomics Institute (Shenzhen, China).

Clean reads were selected using a custom Perl script that removed low-quality sequences (more than 50% of bases with quality scores < 20), reads with more than 5% unknown (“N”) bases, and reads containing adaptor sequences. After removal of rRNA sequences from the clean reads, the remaining transcripts were aligned to the *Ae. tauschii* reference genome and used to reconstruct the transcriptome. RNA-seq data analysis was performed using several packages in the JAVA-based client-server system Chipster [29].

### Annotation and normalization of gene expression levels

Gene abundances were quantified with RSEM software [30] and sequencing reads were mapped to the reference sequence (*ta IWGSC_MIPSv2.1 genome D*). First, a set of reference transcript sequences was generated and preprocessed. Second, RNA-seq reads were realigned to the reference transcripts using the Bowtie alignment program, and the resulting alignments were used to estimate gene abundances. Expression levels of all detected genes were obtained by calculating the expression level of this gene with the coverage of reads in one gene.

Gene expression levels were then normalized using the FPKM method according to the following formula:

$$ FPKM(A)=\frac{10^6\mathrm{C}}{NL/{10}^3} $$

where FPKM (*A*) is the expression of gene *A*, *C* is the number of fragments mapped to gene *A*, *N* is the total number of fragments mapped to reference genes, and *L* is the number of bases on gene *A* [31]. The FPKM value can be directly used to compare differences in gene expression among samples, as this method is able to eliminate the influence of different gene lengths and sequencing data amounts on gene expression calculations.

### Analysis of expression patterns

Pairwise Pearson correlation coefficients were calculated between samples based on the expression of all gene sets. Correlations between each pair of samples were then visualized in the form of a correlation heat map using a function in R (http://www.r-project.org/).

After annotation of gene expression levels, identical genes were compared between samples. The FDR was used to determine the *p*-value threshold in multiple tests, with the criteria of FDR < 0.05 and |log_2_ ratio| > 1 used in subsequent analyses to judge the significance of gene expression differences. In the case of more than two samples, the union (or intersection) of all DEGs was used in subsequent analyses. When multiple transcripts mapped to the same gene, the longest transcript was used to calculate the gene expression level and coverage.

### GO and KEGG functional annotation and classification

GO analysis, used to identify the main biological functions of DEGs, is an international standardized gene functional classification system that has three ontologies: molecular function, cellular component, and biological process. To determine their main biological functions, DEGs were annotated with terms in the GO database (http://www.geneontology.org/) using Blast2GO [32] according to their numeric orders in the nr database to determine the main biological functions. Genes usually interact with each other to play roles in certain biological functions. Pathway-based analysis helps to further understand gene biological functions. KEGG is the major public pathway-related database [33]. Pathway enrichment analysis was used to identify metabolic or signal transduction pathways significantly enriched in DEGs compared with the whole genome background. The calculating formula was the same as that used in the GO analysis as the calculated *p*-value adjusted based on a FDR < 0.05 threshold. Pathways meeting this condition were defined as significantly enriched in DEGs.

### Excavation and functional annotation of new genes

Novel genes and transcripts were explored and existing genome annotation information was complemented by comparing the original genome annotation information with the reference genome sequences. New genes were discovered using the annotation library NR [34] as well as KEGG sequence alignment [35] using BLAST [36] software. New gene annotation information was obtained from the homologous gene annotation information in the library.

### RT-qPCR

Total RNA (2 μg) from each sample was reverse transcribed into cDNA using a RevertAid First Strand cDNA Synthesis kit (Thermo Scientific) according to the manufacturer’s instructions, and stored at − 20 °C. A total of 8 selected genes related to selenium were analyzed by RT-qPCR using the SYBR Green qPCR Master Mix (TaKaRa) and the ABI ViiA 7 real-time PCR system (Applied Biosystems). The primer sequences are listed in Additional file 12: Data S9. Relative quantification was calculated by the comparative 2^-ΔΔCt^ method [37]. All reactions were performed in triplicates per sample.

## Additional files

Additional file 1: **Data S2.** Information statistics of reads filtering and HQ clean data compared with rRNA. (XLS 26 kb)

Additional file 2: **Figure S1.** Classification of raw reads in each sample. (TIF 809 kb)

Additional file 3: **Data S1.** Information statistics of data before and after filtering. (XLS 27 kb)

Additional file 4: **Data S3.** Comparison statistics of reads which were unmapped with rRNA compared with the reference genome. (XLS 25 kb)

Additional file 5: **Data S4.** General statistics of detected gene’s number in each sample. (XLS 24 kb)

Additional file 6: **Figure S2.** Correlation heat maps of each sample. (TIF 459 kb)

Additional file 7: **Data S5.** Detailed genes list for each category. (XLS 1856 kb)

Additional file 8: **Figure S3.** Seven genes regulated in a Se-related were verified by RT-qPCR. (TIF 204 kb)

Additional file 9: **Data S6.** GO functional classification of unique DEGs in each group of CK1 vs.T1 and CK2 vs.T2. (XLSX 14 kb)

Additional file 10: **Data S7.** KEGG pathway annotation of unique DEGs in each group of CK1 vs.T1 and CK2 vs.T2. (XLSX 16 kb)

Additional file 11: **Data S8.** Characteristic transcription factor genes list between comparison groups. (XLS 46 kb)

Additional file 12: **Data S9.** List of primers used in RT-qPCR. (XLS 26 kb)

## Abbreviations

DEGs: Differentially expressed genes
FDR: False discovery rate
FPKM: Fragments Per Kilobase of exon model per Million mapped reads
GO: Gene Ontology
HQ clean reads: High-quality clean reads
KEGG: Kyoto Encyclopedia of Genes and Genomes
RT-qPCR: Real-time fluorescence quantitative PCR
TFs: Transcription factors
