# Supplementary figures and images for: Comparative transcriptome analysis of two selenium-accumulating genotypes of Aegilops tauschii Coss. in response to selenium
Source: BMC Genet. 2019 Jan 14;20:9. doi: 10.1186/s12863-018-0700-1 (PMC6332533; doi:10.1186/s12863-018-0700-1)

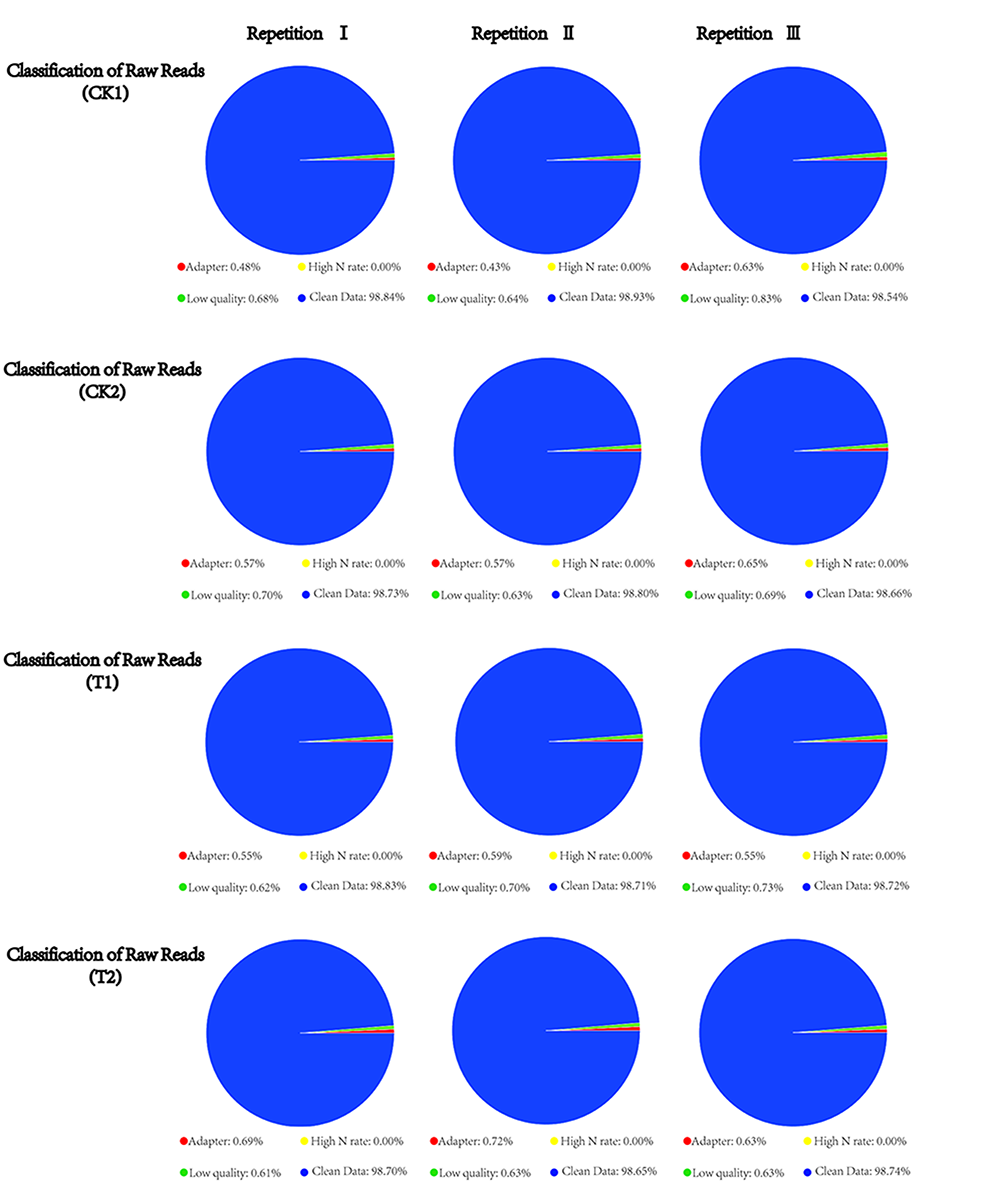

Supplement: Supplementary file 2 — Figure S1. Classification of raw reads in each sample. (TIF 809 kb) [file 12863_2018_700_MOESM2_ESM.tif]

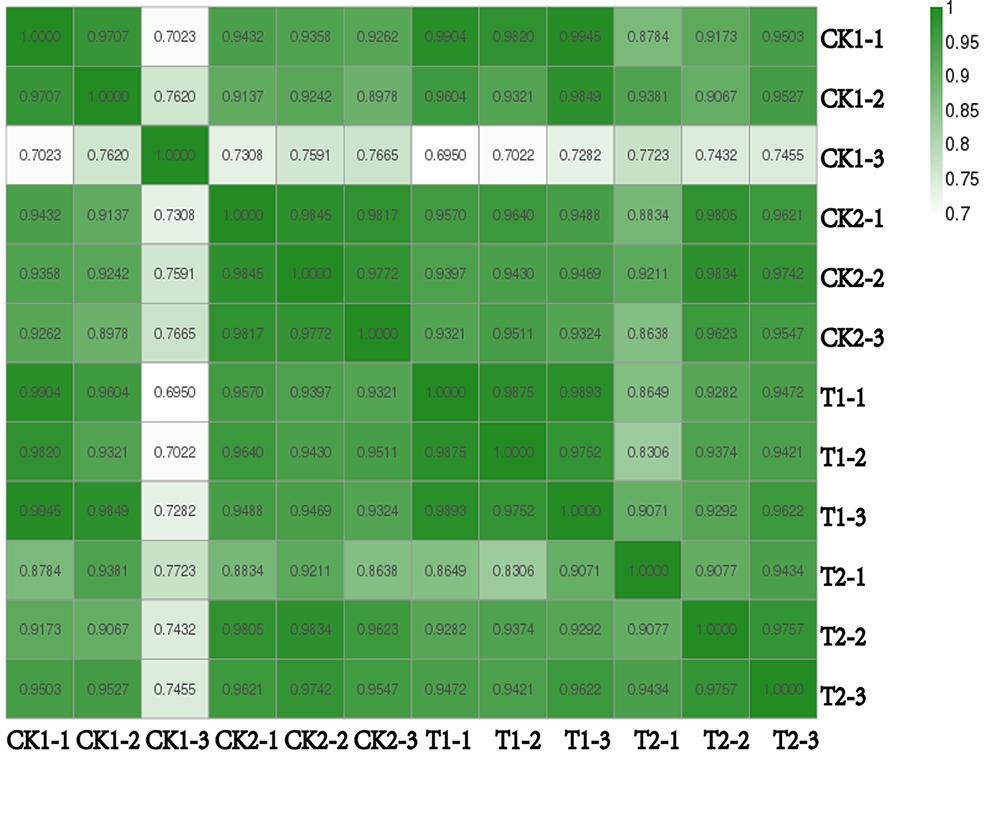

Supplement: Supplementary file 6 — Figure S2. Correlation heat maps of each sample. (TIF 459 kb) [file 12863_2018_700_MOESM6_ESM.tif]

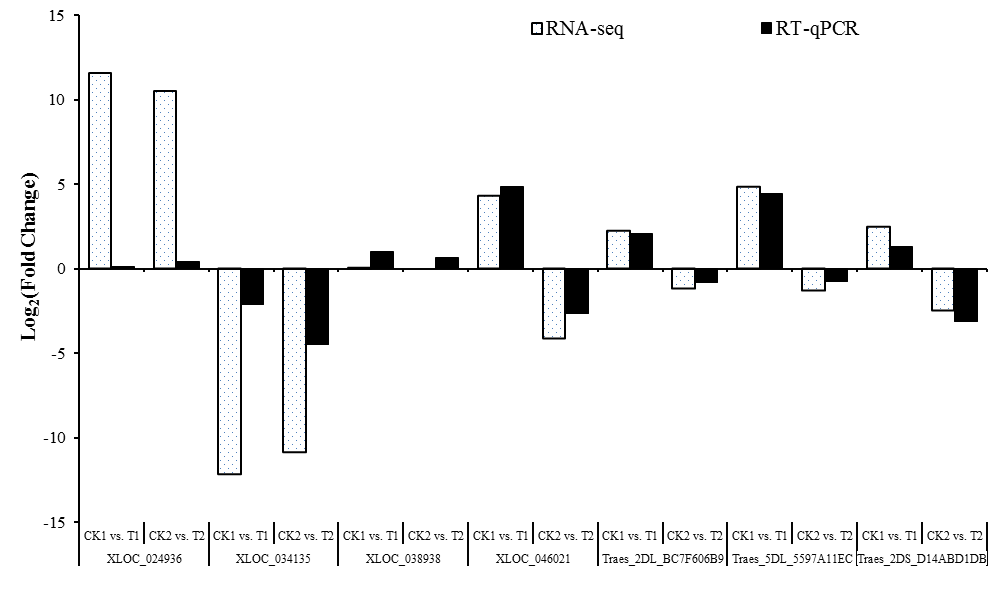

Supplement: Supplementary file 8 — Figure S3. Seven genes regulated in a Se-related were verified by RT-qPCR. (TIF 204 kb) [file 12863_2018_700_MOESM8_ESM.tif]
